# Genomic Approaches for Conservation Management in Australia under Climate Change

**DOI:** 10.3390/life11070653

**Published:** 2021-07-04

**Authors:** Isabelle R. Onley, Katherine E. Moseby, Jeremy J. Austin

**Affiliations:** 1 Australian Centre for Ancient DNA (ACAD), School of Biological Sciences, University of Adelaide, Adelaide, SA 5000, Australia; jeremy.austin@adelaide.edu.au; 2 Centre for Ecosystem Science, School of Biological, Earth and Environmental Sciences, University of New South Wales, Sydney, NSW 2052, Australia; k.moseby@unsw.edu.au

**Keywords:** conservation genomics, climate change, assisted migration, genetic rescue

## Abstract

Conservation genetics has informed threatened species management for several decades. With the advent of advanced DNA sequencing technologies in recent years, it is now possible to monitor and manage threatened populations with even greater precision. Climate change presents a number of threats and challenges, but new genomics data and analytical approaches provide opportunities to identify critical evolutionary processes of relevance to genetic management under climate change. Here, we discuss the applications of such approaches for threatened species management in Australia in the context of climate change, identifying methods of facilitating viability and resilience in the face of extreme environmental stress. Using genomic approaches, conservation management practices such as translocation, targeted gene flow, and gene-editing can now be performed with the express intention of facilitating adaptation to current and projected climate change scenarios in vulnerable species, thus reducing extinction risk and ensuring the protection of our unique biodiversity for future generations. We discuss the current barriers to implementing conservation genomic projects and the efforts being made to overcome them, including communication between researchers and managers to improve the relevance and applicability of genomic studies. We present novel approaches for facilitating adaptive capacity and accelerating natural selection in species to encourage resilience in the face of climate change.

## 1. Introduction

In the time since European arrival in Australia, native plants and animals have suffered major population decline and extinction. Ten percent of endemic mammal species known to be present in the 18th century are now extinct, and many others survive only on offshore islands and fragmented habitat [1]. Further, some 38 species of vascular plants, 10 invertebrates, 4 frogs, 3 reptiles, 1 fish, and 9 bird species have been confirmed extinct since European arrival in 1788 [2]. These impacts have been attributed to a number of factors, most notably land management changes (including land clearing for cropping and grazing), alterations to fire regimes, and the introduction of feral predators, including cats (*Felis catus*) and red foxes (*Vulpes vulpes*), and feral herbivores such as European rabbits (*Oryctolagus cuniculus*) [2,3,4]. However, extinction risk is being further exacerbated by human-induced climate change [5], with rapidly warming temperatures and increased frequency and magnitude of extreme weather events such as drought and fire resulting in phenological shifts, range contractions, and population declines in many taxa [5,6,7].

Since the late 20th century, the importance of genetic factors in the science of conservation biology has been well recognised; inbreeding depression and loss of genetic variation have been identified as potential drivers of extinction [8,9]. For example, an isolated population of mountain pygmy possums (*Burramys parvus*) at Mount Buller in Victoria suffered a considerable loss of fitness following a rapid population decline and subsequent inbreeding [10]. Processes such as inbreeding and genetic drift, particularly in threatened species with small, isolated populations, can result in a high frequency of deleterious alleles, exacerbating extinction risk [11]. With this knowledge, genetic analyses are now a vital part of conservation biology in Australia [12,13,14], with several approaches currently being considered as potential strategies for maintaining, and in some cases increasing, the genetic diversity and resilience of threatened species [14,15].

With the advent of advanced DNA sequencing technologies, it is now possible to approach management of threatened species under a changing climate at the genomic level, taking into account not only genetic diversity and inbreeding effects, but fitness, gene expression, and adaptation [16,17]. The relevance and application of genetic tools to conservation have been discussed extensively in the literature [11,18,19,20], as have the various genomic approaches for DNA sequencing and analysis [21,22,23]. Here, we focus specifically on genomic approaches to conservation management under climate change in Australia—a continent with a range of climate change challenges, large latitudinal and environmental gradients, and a biota that has already suffered disproportionate rates of extinction, population fragmentation, and decline. However, the challenges presented by climate change to conservationists and the potential solutions discussed herein are applicable on a global scale. This review aims to discuss the current and expected conservation challenges associated with anthropogenic climate change, followed by the progress of conservation genomics to address these challenges. We explore some of the issues surrounding the application of such technologies to conservation and management strategies and highlight emerging opportunities to apply genomics to conservation in Australia.

## 2. Climate Change and Conservation Challenges

Anthropogenic climate change has caused Australia’s average temperature to increase by 1 °C in the last century, and further warming is expected [24]. By the year 2090, annual average temperatures may rise by 5 °C [25]. Climate change has also been linked to an increase in extreme weather conditions [26], including more frequent and intense bushfires [27], cyclones [28], and floods [29]. These rapidly changing conditions compound the existing threats from habitat loss, fragmentation, feral predators, and competitors and are exacerbating extinction risk, all of which present new and pressing challenges for conservationists [5,6,7,30]. Two of the most critical issues relate to species’ ability to shift their range or adapt in situ. While species may once have undergone range shifts in response to changing climates during the Late Pleistocene and Holocene [31], habitat loss and fragmentation are likely to hamper this response in the majority of species, particularly those with short dispersal distance. In the face of rapidly changing climate many species may not be able to adapt in situ due to low standing genetic variation, reduced gene flow, and/or limited phenotypic plasticity [32,33].

The initial consequences of climate change for Australian flora and fauna have been well documented in recent years and include range shifts, population declines, altered migration rates, and altered selection pressure [34,35,36,37,38]. Changes to the physical environment have resulted in catastrophic cascading ecosystem effects and negative feedback loops [39,40]. The impacts of climate change are evident across a range of habitats and environments, from the oceans [41] to the tropics [42] and even into the arid and alpine zones [43]. Montane species are being forced into higher altitudes as temperatures increase and will inevitably be forced “off the mountain top” [44]. Species with specific habitat and climatic tolerance ranges are predicted to be vulnerable to rising temperatures [45]; mechanistic models of future climate conditions predict a reduction in reproductive output of green sea turtles (*Chelonia mydas*) associated with marine heatwaves [46]. Conradie et al. [47] predict that by 2100, zebra finches (*Taeniopygia guttata*) will be exposed to acute lethal dehydration risk for several weeks of the year in over 50% of the species’ current range. Climatic extremes have already resulted in massive diebacks of mangroves [48] and seagrass [49]. Furthermore, less resilient species with specific habitat requirements are becoming increasingly vulnerable due to shifts in their climatic niche. For example, only 30% of the current distribution of *Banksia marginata*, a highly fragmented but ecologically significant plant species, overlaps with the projected distribution under climate change by 2080 [50].

Unfortunately, despite these threats, the vast majority of management plans for threatened species do not currently include actions to improve adaptability to climate change [51].

## 3. Conservation Genetics in the Genomics Era

Conservation genetics is a discipline that incorporates genetic information into the planning and management of threatened species to minimise extinction risk. Genomic measures of relatedness, connectivity, and differentiation can be applied in a broad context to identify and clarify taxonomic issues and to identify evolutionarily divergent lineages within species [52]. At a local level, conservation managers can use genetic information to monitor gene flow and landscape genetics, as well as population parameters such as heterozygosity, genetic drift, and levels of inbreeding [14,53]. Genetics has also been used to inform pedigrees and breeding programs for endangered species in captivity by determining factors such as individual fitness and kinship [18,54,55].

Recent developments in high throughput DNA sequencing and its application to genomics have made genetic analysis more advanced and affordable for researchers [56]. Since 2005, DNA sequencing costs have reduced 5-fold, and the number of genetic markers available has increased by at least 2–3 orders of magnitude [57]. These genomic methods utilise high throughput sequencing technologies to sequence millions of DNA fragments in parallel, allowing thousands of genetic markers to be sequenced from hundreds of individuals in a single assay [58,59]. Previously, sequencing of mtDNA or nuclear genes or analysis of microsatellite loci limited genetic analyses to one to tens of loci and focused almost exclusively on neutral (or nearly neutral) loci [60]. While traditional methods were effective for taxonomy, phylogeography, and population genetic studies, genomic sequencing allows conservation geneticists to generate and analyse large data sets that include neutral and functional loci. The ability to assay functional variation extends the focus of conservation genetics to include processes such as natural selection and adaptation and to examine the fitness consequences of inbreeding [61,62]. Geneticists can now sequence the entire genome, use exome capture to target specific regions, or target single nucleotide polymorphisms (hereafter, SNPs) [63]. Although the massive amounts of data produced by genomic sequencing platforms necessitate advanced and diverse bioinformatics tools [58,64], such programs are constantly being improved and developed to allow genomic sequencing to reach its full potential. While there are still some uncertainties surrounding interpretation and uptake of genomic data in a management context [56], population genomics studies are increasingly being applied to conservation problems and management decision-making [65]. Genomic data have already been used to extensively study and characterise the genetic diversity of Australian wildlife, including quantifying the genetic effects of translocations in small mammal populations and identifying candidate genes associated with breeding success in marsupials [14,66,67,68,69,70].

The advances in genomic sequencing methods have made it an invaluable tool for conservation biologists, particularly when studying selection, adaptation, and functional diversity in threatened and economically valuable species [71,72]. For example, genomic studies of the Tasmanian devil (*Sarcophilus harrisii*) by Epstein et al. [73] revealed signals of selection in genes associated with immune function or cancer risk in three populations decimated by facial tumour disease, likely the result of an evolutionary response to the illness. This discovery has the potential to inform future selective breeding in the species, enhancing the prevalence of these resistant genotypes in insurance populations for the ongoing persistence of Tasmanian devils. SNP analysis of commercially important abalone (*Haliotis rubra*) identified genotype associations with several variable aspects of marine habitat, including sea surface temperature and ocean current, providing important insight into species resilience under fluctuating marine climates [74]. Genomic sequencing has also been used to identify local adaptation in gimlet trees (*Eucalyptus salubris*) [75] and potential selection in response to sea surface temperatures in seaweed (*Phyllospora comosa*) [76]. SNP genotyping performed on degraded samples seized from the wildlife trade has even been used to identify population structure and differentiation of threatened species [77].

## 4. Application of Conservation Genomics to Climate Change Challenges

**Figure 1:**
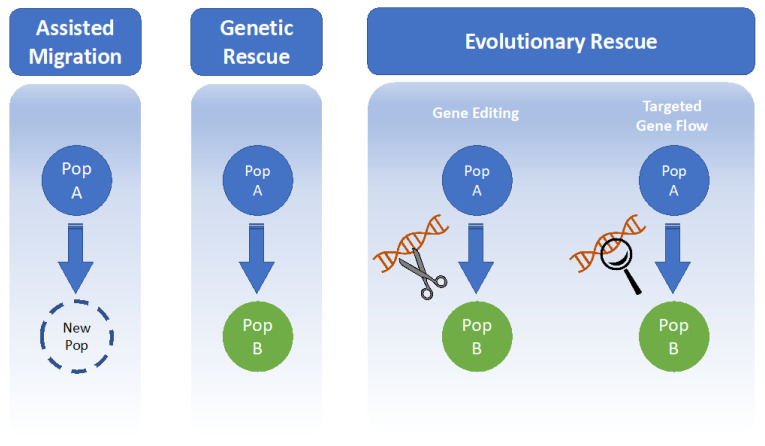
A summary of the conservation approaches discussed in this review that may be informed by genomics.

Genomics can provide critical new data to inform conservation management of threatened species under climate change in two key ways. Neutral variants—changes to the DNA sequence that have no effect on the viability of the individual—can be analysed to understand population processes such as gene flow, changes in population size, and population structure. Meanwhile, functional variants—DNA sequence changes that have fitness consequences—can be analysed to identify genetic diversity and patterns of local adaptation across potential source populations. Such knowledge may contribute to facilitating assisted range shifts, identifying suitable source populations for translocations and restoration carrying genotypes adapted to conditions at the recipient site [15], and enhancing local adaptation to climate change stress. An important application of conservation genomics is to inform species translocations, the facilitated movement of a species to an area within its historical range or to a new location with a suitable current or projected climate and habitat [78]. Traditionally, conservation managers conduct translocations to establish insurance populations, increase population size, and encourage heterogeneity [79,80]. However, Sgro et al. [81] argue that translocation should be considered not only as a method of increasing population sizes in threatened species but also as a means of creating “evolutionary resilience” to climate change. Assisted migration and genetic rescue are types of species translocation that may have the potential to offset the effects of climate change [82,83]. Furthermore, evolutionary rescue via processes such as targeted gene flow, another type of translocation, and gene editing, the process of altering DNA coding sequences to remove deleterious/insert advantageous alleles, may provide conservation solutions in the face of anthropogenic environmental shifts by quickly and efficiently improving the resilience of a population to external stressors [84,85,86]. These techniques are summarised in Figure 1. It is important to note that many of these technologies and approaches are still in the early stages of development, and while their potential uses are promising, limitations remain that are discussed further in subsequent sections of this review.

### 4.1. Assisted Migration

Assisted migration (or assisted colonisation) is the intentional movement of species to areas where habitat is predicted to become suitable as the climate changes (Figure 1) [87]. This usually refers to translocation of individuals outside their historical range but may include reintroductions to climatically suitable locations within the former range for species that have suffered large historical range contractions. Due to habitat fragmentation, many species that once encompassed large ranges no longer exist along an environmental gradient or have the capacity to disperse in response to climate change threats and stressors. In such scenarios, assisted migration may prove effective, particularly for sessile species or those with low dispersal ability [88].

Gallagher et al. [82] summarised the traits associated with species most likely to be affected by climate change and in need of assisted migration. Of most relevance to genomic applications to conservation are species with reduced adaptive capacity (poor ability to evolve in situ or disperse), small effective population size, and reduced genetic diversity. These features may be a result of recent population declines, long term effects of narrow ranges (narrow endemics) or niche specialisation, meta-population structure (new or existing barriers to gene flow), and distribution (for example, species in the tropics may have less adaptive capacity for temperature stress due to limited thermal seasonality). Examples of assisted migration outside a species’ historical range are rare; however, Supple et al. [89] examined genomic variation in remnant populations of critically endangered yellow box (*Eucalyptus melliodora*) to inform restoration plantings in this species that has been reduced to less than 5% of its original range. By combining genomic data with environmental variables and climate predictions, they were able to identify sites for assisted migration and suitable source populations containing genetic variation adapted to future climate predictions.

### 4.2. Genetic Rescue

Translocation may also be used as a method of genetic rescue, whereby new individuals (and subsequently new genetic material) are introduced into an existing population with the aim of increasing population fitness and adaptive potential by increasing heterozygosity and adaptive capacity, masking deleterious alleles, countering the effects of inbreeding depression, and reducing genetic load (Figure 1) [15,83,86,90,91,92,93]. A well-known example of genetic rescue involves the mountain pygmy possum (*Burramys parvus*); an isolated population at Mount Buller was supplemented twice with genetically divergent males from larger populations, resulting in increased fitness and fecundity in the subsequent hybrids [10]. Genetic rescue can be applied to any taxa; experimental crosses between populations of a rare perennial daisy (*Rutidosis leptorrhynchoides*) resulted in similar or increased levels of heterosis across three generations [94]. Advances in genomics have given managers the ability to refine the science of genetic rescue further by testing for the presence of inbreeding depression in target populations, to predict the likelihood of gene flow causing outbreeding depression, to identify adaptive variation, and to closely monitor the results of population admixture for genetic rescue [95,96]. Emerging genomic technologies may even be used to predict the fitness consequences of alleles in a population, although some uncertainty remains around this method [93]. Genetic rescue is likely to become increasingly important under climate change, particularly given the tendency for environmental stress to increase inbreeding depression [97,98].

### 4.3. Evolutionary Rescue

A more specific variation of genetic rescue is evolutionary rescue, wherein adaptive evolutionary change is introduced to a population rather than overall genetic diversity [84]. One method of evolutionary rescue is targeted gene flow, a form of translocation that involves the introduction of new individuals with particular traits into an existing population with the aim of increasing a population’s evolutionary resilience (Figure 1). In terms of climate change threats, individuals from a population with favourable alleles, e.g., resilience to high temperatures, could be translocated to another population of the same species that is not adapted to the threat, thereby increasing the resilience of the overall population within a few generations [99]. An example of how targeted gene flow can enhance evolutionary resilience was presented in a pioneering study by Kelly and Phillips (2019) [100], who suggested that the introduction of northern quolls (*Dasyurus hallucatus*) that avoided eating poisonous and invasive cane toads (*Rhinella marina*) to a quoll group naïve to the risks of eating the toads could result in a rapid adaptive response and, ultimately, a more resilient population. Hybrid offspring of toad-exposed and toad-naïve parents showed similar phenotypic responses to offspring of toad-exposed parents only, suggesting the presence of a dominant heritable trait for “toad-smart” behaviour. Although yet to be tested on a real-world population, the results of this study indicate that it is possible to introduce an adaptive response to a threat in a population through targeted gene flow. For targeted gene flow to be successful, however, knowledge of trait variation, heritability, and the underlying genetic variants linked to the trait are needed in order to identify suitable individuals to translocate.

Within a single species, certain populations may be better adapted to environmental stressors than others. For example, genomic sequencing has revealed within-species variation in heat stress response in both animals and plants [101,102,103]. This has important implications for species management under climate change. Recently, Cummins et al. [104] used the commercial genomic sequencing platform Diversity Arrays to conduct a genome-wide analysis of the Australian crawling frog (*Pseudophryne guentheri*), which revealed signals of local adaptation and limited gene flow between populations. While individuals living in the hotter, drier regions of the species’ range were better adapted to predicted conditions in Australia under climate change, the more mesic individuals were not. Similarly, a study on greenlip abalone (*Haliotis laevigata*) revealed adaptive divergence across ~800 km of coastline that was strongly linked to minimum sea surface temperature and oxygen concentration [105]. In both cases, targeted gene flow between populations may encourage viability in the face of rising temperatures and other environmental shifts associated with climate change. Varied resilience to high temperatures has also been observed in coral reefs across natural temperature mosaics, with corals from warmer locations exhibiting mild selection in response to heat stress events [106,107]. A recent study by Quigley et al. [108] modelled the spread of temperature tolerant loci in corals in the Great Barrier Reef under natural and assisted scenarios. They concluded that adaptive variants are unlikely to spread fast enough to combat current rates of warming without human intervention. Targeted gene flow has therefore been flagged as a potential strategy to combat coral bleaching under climate change [109]. Further, Jordan et al. [110] identified 81 adaptive SNPs in the genome of mottlecah trees (*Eucalyptus acrocarpa)*, many of which were associated with variables of aridity, temperature, and rainfall, while Steane et al. [111] studied the genomes of a forest tree species, *Eucalyptus tricarpa*, across an area encompassing significant variation in aridity. Genomic divergence was found to be strongly correlated with temperature and moisture availability, evidence of local adaptation to environmental stressors associated with climate change predictions. The authors suggest that such information on the adaptive capacity of the species could be used to inform assisted migration in order to fix beneficial alleles and safeguard vulnerable populations against climate change.

Another underexplored genetic approach to addressing climate change impacts through evolutionary rescue is gene-editing. Already used extensively in agriculture, gene-editing involves the use of functional proteins to target a location in the genome and alter the gene’s coding sequence or activity (Figure 1) [112]. Commonly, the RNA-guided Cas9 enzyme (isolated from CRISPR acquired immune systems in bacteria) is used to target and cut the DNA sequence, enabling insertion, deletion, and replacement [113]. Once considered impractical for wild populations, gene-editing technology has recently become much more accessible to conservation biologists [114]. Although research to date has focussed predominantly on the application of gene-editing to disease prevention and the suppression of invasive species, with the new capacity of genomic sequencing technology to identify adaptive alleles associated with environmental stressors [115], it follows that the isolation, introduction, and fixation of these in a population would be possible via gene-editing [116,117].

In particular, CRISPR technology has the potential to be used for gene drives, wherein a beneficial trait is introduced and fixed in a population far more rapidly than natural selection allows [118]. For example, populations of American chestnut trees (*Castanea dentata*) have been decimated by the invasive pathogen chestnut blight fungus (*Cryphonectria parasitica*) since the early 20th century [119]. Researchers recently succeeded in developing transgenic American chestnut trees that demonstrate tolerance to the fungus by inserting a gene from wheat into the genome [117]. Gene editing could also be used to introduce deleterious alleles to populations of invasive species in order to reduce fitness and/or fecundity [114,118]. Johnson et al. [120] champion the applications of gene-editing technology for conservation, including the possibility of removing genetic disorders from a population, increasing genetic diversity following a bottleneck, or controlling the spread of invasive species. It represents a method of introducing beneficial alleles to a population that is threatened by climate change, particularly in situations where translocations are not possible [112]. In some systems, such as coral reefs, the introduction of natural or synthetic genes may aid in increasing resilience of species vulnerable to climate change effects [121]. Zafar et al. [122] discuss the possibility of using CRISPR technologies to develop novel quantitative trait loci in plants to increase resilience to abiotic environmental stressors including drought, temperature, and salinity. Further, CRISPR microinjection performed on larvae of the reef-building coral species *Acropora millepora* resulted in a ~50% mutation rate on all three target genes [123]. All target genes were putatively responsive to environmental stressors.

## 5. Overcoming Barriers to the Application of Genomics for Conservation Management under Climate Change

There are some barriers to the application of conservation genomics to management practices in Australia. A detailed discussion of the technical challenges associated with population genomics is beyond the scope of this paper (but see [86,124,125])—here, we aim instead to highlight the difficulties associated with the implementation of conservation genomics in management and how they can be overcome. First, the link between research and conservation practitioners must be strengthened to allow managers to set goals, make informed decisions, and integrate the findings of conservation geneticists with on-ground management practices in real-time [56,126,127]. A recent survey of 148 conservation practitioners in New Zealand indicated that although collaboration with geneticists was desired, managers did not know how to reach them [128]. Furthermore, Cook and Sgro (2017) [129] highlight the need for increased presence and engagement of evolutionary biologists in the conservation space, while Shafer et al. [56] observe that encouraging genome researchers to communicate directly with practitioners about the decreasing costs and potential uses for genomic technology, as well as its limitations, would be a step towards resolving the disconnect between scientists and stakeholders. Kadykalo et al. [130] identify the need for an interface that allows researchers to engage and connect with conservation managers, who, in turn, may communicate what types of genomic data would be helpful and applicable in the field.

Although many practitioners have been historically averse to admixture as a conservation strategy [131], a cultural shift has recently taken place. There have been a number of cases of successful collaboration between genetic researchers and conservation practitioners in Australia, such as the “devil tools & tech” umbrella framework implemented by the Save the Tasmanian Devil Program [126] and various provenance-related research projects to facilitate ecological restoration [69,132,133]. Indeed, the inclusion of non-academic co-authors in conservation genetics and genomics studies (e.g., [134,135]) has been shown to increase the likelihood of a specific solution- or policy-orientated outcome by up to 250% [136]. Garner et al. [75] note that much of the work occurring in non-academic spaces is not prioritised for publication, but it is clear that a holistic, collaborative approach with open communication and engagement between stakeholders is highly beneficial. Such collaboration not only facilitates the implementation of research findings but also encourages targeted studies that are directly relevant to conservation managers and policymakers and fully utilises the potential of modern genomic technology [137,138].

Second, it must be acknowledged that the application of gene flow and gene editing as management practices carries a certain level of risk. Introducing new individuals to a population may lead to outbreeding depression [139], although the risk of outbreeding depression occurring has likely been overstated, as there is little evidence of its manifestation in wild populations [15,140]. Care must be taken to ensure that deleterious alleles are not being inadvertently introduced to populations and that locally adapted alleles are maintained [88,141]. A recent genetic rescue of Trinidadian guppies (*Poecilia reticulata*) resulted in increased fitness without swamping locally adapted alleles; however, the authors note that the results are not directly transferable to other organisms and that genetic rescue should be considered and planned case-by-case [142]. Furthermore, adaptational lags to contemporary temperature increases may mean that species are not well adapted to the conditions they are currently experiencing within their home-range, necessitating thorough and careful genomic analyses to choose an effective provenancing strategy for assisted gene flow [86,143].

Gene editing is also not without its challenges; Phelps et al. [112] note that while currently used for agricultural enhancement, such an approach would be challenging in a threatened species context due to the complex nature of adaptation and selection in ecology; traits are sometimes driven by a network of genetic responses (i.e., polygenic), rather than a single genomic region [125]. Varshney et al. [144] note that the development of stress-tolerance in crops via gene-editing is difficult, as tolerance can be expressed in many ways and is often the result of many genomic mechanisms. Managers implementing evolutionary rescue in general must also consider that phenotypic expression of genotypes can be unpredictable, and as such, the introduction of a new genotype to a population or area is not guaranteed to have the desired result [86,145,146]. Incorporating phenotypic data into planning strategies may assist in predicting the persistence of species introduced to new environments. Although a significant body of work on risk assessment has emerged in recent times [19,140], there remains a need for more resources surrounding decision-making tools and guidelines for conservation managers hoping to implement conservation genetics in the planning of threatened species management strategies [147]. Careful planning and risk assessment prior to intervention using tools such as those from Rossetto et al. [148] for conservation genomics management workflow are vital if genomic data are to be routinely included in threatened species management. This not only will help prevent undesirable outcomes but also will optimise resource usage and “bridge the gap” between researchers and conservation practitioners [149].

Finally, trust and support from the general public and conservation institutions for the expansion of conservation genomics must be gained in order to provide a solid foundation for future trials and innovation. Some conservation organisations such as zoos have policies against selective breeding that were put in place to safeguard species from becoming oddities or public curiosities [54]. These policies need to be updated to allow their participation in breeding trials and genetic interventions that are conservation focussed. Such institutions also need to play a stronger role in public education regarding genetic interventions. While there are a number of inherent issues associated with captive breeding programs, including genetic risks such as inbreeding depression [150], and behavioural challenges, such as predator naivety [151], breeding establishments such as zoos, herbariums, and seed banks have been identified as potentially vital resources in conservation genomics were their policies to become more flexible, not only as sources of genetic variation and insurance populations but also through providing a controlled environment for hybridisation trials [152,153,154].

## 6. Future Opportunities and Tools to Harness Conservation Genomics in the Fight against Climate Change

Advanced genomic sequencing technology can now be incorporated into conservation management strategies through genomic analyses, targeted gene flow, assisted migration, and gene editing. These methods can all be used in breeding programs, reintroductions, revegetation programs, and translocations to encourage viability in threatened species in the face of rising temperatures and extreme climate events. We see additional opportunities for genomics methods to involve experimental studies and targeted solutions to enable better planning and management for species conservation in the face of climate change. For example, genomic data could be used to determine how phenotypic plasticity and adaptive evolution act within species across environmental gradients in order to predict species’ response and vulnerability to climate change [155]. Climate change experiments, either in the field or laboratory settings, using manipulated climatic conditions and genomic data could be used to identify evolutionary responses to changes in temperature and water availability [156]. This information could then be used to guide translocations and to revise species range loss projections under different climate change scenarios [157].

Accelerating natural selection in response to current and future environmental stressors may be particularly important for the survival of species that have suffered severe range reductions, a common occurrence amongst Australian endemics. Whilst reintroduction programs are becoming common, few take into account future adaptability or, indeed, adaptive capacity of source populations [140,158,159]. Conservation practitioners now need to think seriously about the long-term viability of the populations they are managing under climate change projections. Actions could include maximising evolutionary potential by working towards increased population size, genetic variation, and gene flow in managed populations [86,153] or targeted provenancing strategies involving the selection of source individuals for translocations and reintroductions with an adaptive bias towards predicted climate change conditions [160]. Climate resilience may even be encouraged by exposing individuals to climate stressors, as per Kelly and Phillips (2019) [100]. The greater stick-nest rat (*Leporillus conditor*), for example, is a murid rodent that became extinct on the Australian mainland in the early 1900s, surviving only on a single offshore island [161]. The species became the focus of a number of translocation efforts beginning in the 1980s, including a reintroduction to Arid Recovery Reserve, a 12,300 hectare predator-free enclosure in South Australia’s arid zone [162,163]. Although the translocation was initially considered a success, having retained a viable population for two decades, it was observed that the stick-nest rats demonstrated spikes in mortality during extreme summer heat events [164], a selection pressure that may lead to natural selection for animals with improved physiological adaptations to heat. Comprehensive genomic analyses of the stick-nest rat population at Arid Recovery by White et al. [14] twenty years after the species’ reintroduction identified six loci under putative selection in the genome when compared with founding populations, but further research is required to determine whether these genomic regions are associated with heat stress. This differentiation may be an adaptive response to heat stress experienced during the hot summer months at Arid Recovery, implying that the translocation of greater stick-nest rats has led to the establishment of a population that is better adapted to withstand hotter, drier conditions.

A number of frameworks and guidelines have recently emerged to facilitate the application of conservation genomics and genomic sequencing to wildlife management strategies (e.g., [165]). Hoffmann et al. [153] present a decision-making framework for managers that incorporates the potential and limitations of genomic approaches, as well as guidelines for inferring adaptive capacity and the significance of gene flow in a threatened species population. They note the importance of a robust reference genome (see also [166]) but also acknowledge that this resource is not always essential for detailed analysis of population structure and signals of selection associated with environmental variables, as evidenced by Grabowski et al. [167] and Wood et al. [76].

## 7. Conclusions

With the advent of genomic sequencing, conservation biologists now have the capacity to assess genomic data at a higher resolution than ever before. Not only can overall genetic diversity be analysed but also signals of adaptive evolution, mutations, and inbreeding can now be identified quickly and at relatively low cost. Under a rapidly changing climate, such technology has the potential to revolutionise conservation management; assisted migration, targeted gene flow, and gene-editing can now be performed from an informed perspective, encouraging adaptive capacity and selection for advantageous alleles in threatened populations to improve viability in the face of anthropogenic climate change. Conservation genomics will be of particular value in the management of threatened species with fragmented habitats that are unable to migrate or those with low genetic diversity and limited adaptive capacity. We recommend the application of novel conservation approaches discussed in this review to such taxa in the face of projected climate change. Although such strategies diverge from the traditional in situ conservation paradigm, preservationist methods alone are no longer feasible in the face of widespread climatic shifts. The humbling realisation that, in a comparatively short period of time, humans have induced irreversible changes to the global environment that will be observable in the fossil record for millennia calls for a shift in our attitude toward the world around us [168,169]. As Thomas (2011) [170] notes, “conservation under current circumstances is about managing change; retaining or restoring past community composition is no longer feasible”.

While some limitations remain—species suitability, additional conservation requirements, the risk of outbreeding depression [19,171], and communication barriers between conservation practitioners and geneticists—the potential for conservation genetics utilising genomic sequencing technology must be realised if we are to actively and successfully conserve our remaining biodiversity under the threat of anthropogenic climate change. There are many examples of successful collaborations between researchers, stakeholders, and managers in Australia, such as the Pilbara northern quoll research program, a collaborative monitoring effort between multiple universities, researchers, and Indigenous groups, as well as the Western Australian state government [172] and the Genetic Rescue Project, a network of scientists and stakeholders working towards the recovery of five threatened species (e.g., [135]). Based on the success of these cooperative approaches, we reiterate previous calls [56,126,127,129,130,131] for practitioners and researchers to consider the ongoing genomic viability of species in the face of climate change when planning future conservation actions, to collaborate and communicate, and to harness the wealth of information that genomic sequencing provides for more informed and targeted management strategies moving forward.

## Data Availability

Not applicable.
